# Public health research support through the European structural funds in central and eastern Europe and the Mediterranean

**DOI:** 10.1186/1478-4505-10-12

**Published:** 2012-04-05

**Authors:** Mark McCarthy

**Affiliations:** 1 Department of Epidemiology and Public Health, University College London, 1-19 Torrington Place, London WC1E 6BT, UK

## Abstract

**Background:**

Public health research provides evidence for practice across fields including health care, health promotion and health surveillance. Levels of public health research vary markedly across European Union (EU) countries, and are lowest in the EU's new member states (in Central and Eastern Europe and the Mediterranean). However, these countries now receive most of the EU's Structural Funds, some of which are allocated to research.

**Methods:**

STEPS, an EU-funded study, sought to assess support for public health research at national and European levels. To identify support through the Structural funds, STEPS drew information from country respondents and internet searches for all twelve EU new member states.

**Results:**

The EU allocates annually around €7 billion through the Structural Funds for member states' own use on research. These funds can cover infrastructure, academic employment, and direct research grants. The programmes emphasise links to business. Support for health research includes major projects in biosciences, but direct support for public health research was found in only three countries - Cyprus, Latvia and Lithuania.

**Conclusions:**

Public health research is not prioritised in the EU's Structural Funds programme in comparison with biomedicine. For the research dimension of the new European programme for Structural Funds 2014-2002, ministries of health should propose public health research to strengthen the evidence-base for European public health policy and practice.

## Introduction

Public health, undertaken at organisational and system level through disease prevention and improving the efficiency and effectiveness of health care, contributes importantly to population health and social wellbeing. Evidence is needed to develop public health policies and practice.

Work in a previous EU study, *SPHERE*, described public health research from a European perspective [1]. A strong geographical gradient in publication rates, highest in the Scandinavian countries and UK and lowest in the southern and Eastern Europe, was found for all public health publications and within six sub-disciplinary themes [2]. STEPS (Strengthening Public Health Research in Europe) was developed and funded through the EU's Science in Society programme to investigate the gradient further. The objective of STEPS was to assess the public health research systems in Europe and the contribution of civil society organisations to health research in the EU 'new member states'.

STEPS held workshops at national level in the 12 EU 'new member states' - the ten countries in Central Eastern Europe (Bulgaria, Czech Republic, Estonia, Hungary, Latvia, Lithuania, Poland Romania, Slovakia and Slovenia) and two in the Mediterranean (Cyprus and Malta) joining the EU in 2004 and 2007. The workshops, led by civil society organisations and involving researchers and ministries of health, were focused on national public health research structures and perspectives [3]. STEPS also developed country health research profiles for all 27 EU countries, describing funding of health research and support for universities and national research institutes [4]. The workshops and country profiles showed that all countries have strategies for research overall, but fewer have strategies specifically for health research, nor for public health research [5]. National governments provide most funding, with non-profit foundations; there is no contribution by the commercial sector.

The European Union funds research directly through the European Commission's Directorate for Research and Innovation. In the EU Seventh Framework Programme, 2007-2013, health is a leading research theme, with €1 bn allocated per annum. Within this, one of three funding strands is for public health research, which has supported more than 70 collaborative projects in Europe over the first three years [6].

However, the STEPS country workshops and profiles revealed a second stream of funding from the EU for research - from its Structural Funds. These funds form around a quarter of the EU's budget, and are directed towards support for poorer countries and regions within the Union. The use of the Structural Funds directly for health services has been reported [7] and is the subject of a collaborative study EUREGIO III [8]. The potential for use of the Structural Funds for public health research is investigated here.

## Methods

Public health research was defined broadly as research at health system and organisational levels, including health care, health promotion and health surveillance, differing from biomedical research at laboratory and clinical levels. The European Commission's web sites Erawatch [9] and Pro-Inno Policy TrendChart [10] provides baseline country information (reporting up to 2009 when accessed in 2011) across all areas of research, and with a primary emphasis towards research for technology and business. Information was recorded for the twelve countries joining the European Union in 2004 and 2007.

Students from EU new member states attending the UCL School of Eastern European and Slavonic Studies identified national web page resources about the Structural Funds in their own languages, and provided an initial scoping of the data available. The country partners in STEPS were also tasked to determine the information on the use of the Structural Funds for research, and to provide overview reports. Structures for commissioning health research by country were also available through STEPS [4].

While the Structural Funds were developed within a common template of funding from the European Commission, the 12 new member states each record the information in different ways, in different categories and with different levels of detail. Therefore, further searches were made of national web pages using Google Translate. The findings of these searches were summarised and brought together for comparison.

## Results

The European Union's budget, comprising just over 1% of total GNP of the member states, is allocated in two major tranches - 43% for 'natural resources' (mainly the common agricultural policy), and 37% for 'cohesion policy'. (The budget for research, education and training is around 7% of the total, and for the Health Directorate only 0.07%.) In the period 2007-2013, the EU has allocated most of the Structural Funds to the 12 EU 'new' member states, which have levels of GDP less than 75% of the EU average [11]

There are three funds for Cohesion Policy [12]. The European Regional Development Funds (ERDF) are "helping regions to anticipate and promote economic change through innovation and the promotion of the knowledge society". The European Social Funds (ESF) are "strengthening competitiveness and employment by...investment in human resources, the development of qualifications and competences, [and] the dissemination of information and communication technologies". The third, much small fund, Cohesion Funds (CF) contributes in the same ways as ERDF and ESF, and provides enhanced support for the smaller countries.

Allocations within the Structural Funds are negotiated by each country with the European Commission's Directorate for Regions. The Commission sets the total sum (mainly based on population), reporting structure (Operational Programmes) and broad priorities (which included R&D), while member states determine the balance of these resource against their own priorities. Funds are allocated in the European Regional Development Funds for 'physical' infrastructures, and in the European Social Fund for 'human' resources. Allocations in the Operational Programmes that support research included university and science buildings, studentships and other training, and formal research calls.

### How much funding? how is it used?

The Structural Funds are spent on programmes devised and implemented by the member states, not centrally by the European Commission. However, country programmes are agreed with the Commission, and follow EU policies. Over the full period 2007-2013, around €86 billion, almost 25% of the total, is described as directed to research and innovation [13]. Of this, €50 billion is for "R&D and innovation in the narrow sense" - including €10 billion infrastructure, €9 billion for investment in firms, €6 billion each for R&TD research centres, assistance to SMEs, and improvement of networks, €5 billion in developing human potential, and €3 billion for SMEs environmentally-friendly products and processes.

A request for information directly to the European Commission received the following reply: "Concerning the rate of implementation of this €86 billion, the latest available figures (Annual Implementation Reports 2009, provided by the Member States in July 2010) show that about € 30 billion have been allocated to specific projects (out of which €16 billion for R&D&I in a narrow sense). The remaining part can be committed before the end of the 2013. Moreover, about 3% of the €30 billion (and 5% of the €16 billion) have been allocated to specific projects in the area of Human Health." [correspondence to author]

Little information is published by the Commission on the actual use of the Structural Funds in the current programme, except gross expenditures. On the web page for Cohesion Policy [14], there is a drop-down menu for Projects. The 12 EU countries have together 56 projects listed, across engineering, technology and life sciences. Five projects listed are health-related, including organisation of cross-border care (Czech Republic), an ambulatory care centre (Slovakia), breast screening (Poland), support for pharmaceutical information (Hungary) and a molecular genomics centre (Malta). However, the first two are service developments rather than research, and the last three are laboratory technology rather than public health research.

### Use for health research in new member states

STEPS used national web pages to identify the use of Structural Funds for health research in the EU new member states. The majority of these 12 countries had general R&D levels well below the EU average at the beginning of the period, at 0.6% of GNP or less, although two countries, Czech Republic and Slovenia, at 1.6% were closer to the EU's average level of 1.9%, with Hungary, Estonia, and Lithuania at between 1% and 0.8%. The overall allocation of the Structural Funds is strongly influenced by the population size of the country - Poland receives a quarter of the total, and small countries gain much less. However, the information available from the larger countries is less satisfactory than in some smaller countries, and the other countries provide important examples of alternative approaches.

Some countries, eg Estonia, Latvia, Lithuania and Slovakia, stand out in using the Structural Funds actively for research, and a few have developed competitive research calls from their funds. Some countries have put funding primarily into 'centres', eg Slovenia. And in some countries, for example Malta and Slovakia, there is evident use of the ESF human resources funds to promote research capacities, with masters, doctorate and post-doctorate programmes. Bulgaria, by contrast, has apparently no direct investment for research, although innovation could be supported in "R&D institutions and organizations, municipalities, private or public bodies including NGOs".

The funds are administered through Operational Programmes in very different ways [Table 1]. A minority of countries use their existing research management institutions - for example, Cyprus placed its Structural Funds for research in the Research Promotion Foundation. In contrast, Malta placed €20 m for a molecular genetics centre within an agency working under the Ministry of Finance. Often it was not clear which organization is managing the funding. While in the main the allocations were not identified to academic fields, in a few countries there were developments of biomedical centres with capital costs - for example, Czech Republic proposes a molecular biology centre outside Prague of €100 m. Only in one country, Lithuania, was there evidence of research for public health research, under the title 'administrative capacity and efficient public administration'

**Table 1:** Research and the Structural Funds in the 12 EU new member states

Comment	Research and the Structural Funds
**Bulgaria** Very limited activity for health research	Bulgaria has a low level of research funding, 0.50% of GDP (2008). The seven Operational Programmes do not directly identify R&D. The fourth, Competitiveness, Axis 1.2 (€75 m) includes 'pro-innovative infrastructure', 'pro-innovative services and 'innovation networks' - with beneficiaries "R&D institutions and organizations, municipalities, private or public bodies including NGOs". Also in April 2009, Operational Programme "Regional Development" allocated €17 million for support/upgrading of universities.

**CYPRUS** Actively using SF for research programmes, including (small) public health research	Cyprus has low expenditure on R&D at 0.49% (2009). Cyprus has three private universities and no medical school (although one is developing in North Cyprus). Of €83 million R&D funds, 45% (€38 m) was directly from government funds, €19 m from the universities' budgets, €10 m from abroad (including €8 m from EU) and €16 m from the private sector (€5 m pharmaceuticals). Cyprus has €640 million Structural Funds. RTDI has been implemented through the Cyprus Research Promotion Foundation. There have been two National Research Frameworks (DESMI) - in 2008 €48 million, and for 2009-2011 €40. These were allocated: €33.4 m for natural sciences, €16.6 m social sciences, €14.6 m engineering and technology, €10.2 m agricultural sciences, €5.1 m humanities, €3.2 m health and biological sciences (including public health research). Other State support for biomedical research includes €5 million annually for Cyprus Institute of Neurology and Genetics (University of Nicosia).

**Czech Republic** Strong R&D programme, including biomedicine but not public health research	Czech Republic has medium level investment in R&D at 1.6% of GDP, with public sector investment 38% and private sector 62%. The Operational Programme Research for Development and Innovation has €2 billion, which includes €685 million (33%) for equipment and infrastructure, €685 million (33%) for R & D institutes focused on applied research, strengthening their cooperation with industry (including hospitals) according to the needs of the region, €414 million (20%) for universities' infrastructure of laboratories and IT, and €72 million (3%) monitoring of projects and programmes, studies and analysis, programme publicity, and training and consultancy services. A further operational programme for universities and Academies, funded through the Ministry of Education, Youth and Sports, provides €154 million institutional support. The programme Call 1.2 (2009) for Regional R&D centres had 18 successful applications, predominantly in technical engineering. In Nov/Dec 2010, biomedicine has been favoured with a €100 million molecular genetics centre at Vestec near Prague for Charles University (the project coordinator is the former President of the Academy of Science) and a €12 million Regional Centre of Applied Molecular Oncology at Brno.

**Estonia** Strong SF investment in R&D infrastructures and research programmes. No public health research.	R&D in Estonia has grown from 0.6% of GDP in 2000 to 1.4% in 2009. Spend is 39% natural sciences, 19% engineering, humanities, 15% medical and health sciences, 12% humanities, 9% social sciences, 5% agricultural sciences. Over 2007-2013, Estonia receives 3.4 billion - European Regional Development Fund support of €1.86 billion, Cohesion Fund €1.15 billion euros, and European Social Fund €390 million. ERDF supports €306 million for infrastructure and development of institutions, small-scale research equipment, R&D in biotechnology and other targeted programmes, and international collaboration. Operational Programme for Human Resource Development, operated by the Estonian Ministry of Education and Research receives €102 million euros (plus €14 m state co-funding). Programmes include Mobilitas, supporting postdoctoral research (€20 million euros), implemented by the Estonian Science Foundation (July 2011 there had been five rounds of calls for 'top researcher grants'), and state's Archimedes Foundation funding internationalization of doctoral studies (€32 million).

**Hungary** Strong use of SF for R&D. Support to industry and higher education institutes. No focus for public health research.	There is a low rate of research investment in Hungary at 1% of GNP. The Structural Funds (€22.4 billion) are allocated through 15 Operational Programmes of the National Development Plan. RTDI activities are mainly supported under the Economic Development Operational Programme (EDOP). Priority 1 "R&D and innovation for competitiveness" has €822 m over 7 years from ERDF for three fields: the promotion of market-oriented R&D; innovation clusters and technology parks; and R&D activities by enterprises. The Social Infrastructures Operational Programme supports research and educational infrastructure at HEIs, and the Social Renewal Operational Programme for collaborative RTDI, including basic research. Together with EDOP, these have more funds than the main national Research and Technological Innovation Fund.

**Latvia** SF used actively for infrastructures and R&D calls. Small public health research support.	Latvia has a low rate of research, 0.61% of GDP (2008). In the ESF operational programme Priority "Higher Education and Science" includes "Attraction of Human Resources to Science" (€47 m) and "Support to Doctor's and Master's study programmes" (€58 m). But many of the scheduled activities were cancelled with the financial crisis. In the ERDF 2.1 Priority "Science and Innovation" has €451 m ERDF support. This includes "Science, Research and Development" for investigator-initiated proposals (€50 m). In a call in 2010, from 177 proposals 114 were financed. A second activity "Development of the scientific and research infrastructure" covers infrastructural development in 10 National research centres and the development of scientific computing network with total ERDF support €175 m. Among these 10 centres is the national research centre in public health and clinical medicine. In the 3^rd ^operational programme "Infrastructure and Services" (ERDF/CF), €168 m is given to development of infrastructure for higher education, including large equipment. Activity "Development of Science and Technology park of Riga", originally intended to support biomedical research, has been put on hold because of absence of suitable land for development, as a consequence of privatisations.

**Lithuania** Strong use of SF for research - industry, human capacities and programmes. Strong list of public health research support.	Lithuania has a relatively low level of research at 0.82% of GDP in 2007. The annual Structural Funds for Lithuania 2007-2013 are €1 bn, around 15% of the total national budget. Planning with stakeholders was developed from 2005. Support for research and innovation is well-developed under all three priority areas. Operational Programme 1-3: 'Enhancement of researchers' capacities', coordinated by the Ministry of Education and Science, includes development of scientists and researchers, thematic networks and R&D training (€140 m). Operational programme 2.1: 'R&D for competitiveness and growth' has €602 m, includes infrastructure projects, 'high level research centres', business parks and integrated studies Operational Programme 3.2: Priority 1.4 Strengthening of Administrative Capacity and Increase of Efficient Public Administration (€178 m) includes Priority 1-4.3 (€37 m), which integrated science, study and business centres (valleys), joint research programmes, strengthening the Lithuanian Scientific Council, and the development of monitoring of science and studies. Research supported by this last measure include the analysis of public health care carried out by municipalities, studies to identify the scope of public health services, the development of a monitoring system, creation of models for providing public health services, training and professional development of public health care specialists, creation of a demand planning system as well as improvement and development of public health impact assessment.

**Malta** Moderate use of SF for public sector research infrastructures and human resources, but not public health research.	In 2007, Malta spent 0.6% of GDP on research and development. Business contributed the largest proportion of funds with €21 m (65%) (largely multinational firms undertaking in-house R&D), followed by higher education €10 m (31%), with public research organisations just €1 m (3.3%). Malta's Structural and Cohesion Funds for 2007-2013 total €855 m. Just under 10% is allocated to 'Knowledge & Innovation', mainly for infrastructures (eg the IT faculty at the university, strengthening university laboratories in engineering, biotechnology and chemistry, €49 m). Malta Enterprises, an agency working under the Ministry of Finance, receives €20 m for a Life Sciences Centre (molecular genetics). An Educational Pathways Scholarship Scheme for Post-Graduate studies (MSc, PhD) is established with €10 m, and Centre for Policy Research and Training for the Public Sector €3.4 m.

**Poland** Very large overall SF available, smaller proportion for R&D, focus on technologies, no public health research	The research expenditure in Poland is low (0.61% GDP in 2008), mainly non-competitive public funding through a large number of higher education institutes and academies. The Structural Funds for Poland 2007-2013 at €67.3 billion are the largest for any member state, allocated in four main programmes. The smallest, the Operational Programme Innovative Economy, with €8.85 billion from ERDF, has two research-related programmes: 'Research and development of new technologies' (€1.1 billion) covering informatics, technologies and biotechnologies (includes 'new medical products and techniques'). The Operational Programme Infrastructure and Environment (€27.9 billion) includes Priority 12 'Health security and improving the efficiency of the health system' (€350 m from ERDF), although this is not related to research. Priority 13, 'Infrastructure of higher education' (€500 m from ERDF) covers infrastructures, access and improving the quality of education through IT. In the Human Capital Programme (€9.7 billion) is Priority 4.2, 'Developing R&D staff qualifications and increasing awareness of science importance to economic growth' (€61 m.). Regional Operational Programmes (€16.6 billion) have been created for each of the 16 provinces. Some Regional Innovation Strategies include innovation networks and R&D.

**Romania** Substantial SF funding, moderate use for research infrastructures and human resources, and competitive programme calls include 'health' but not public health.	Romania has a low level of R&D investment (0.58% GDP in 2008). There is growing use of competition in public funding of research, and of the structural funds to support research, but substantial public budget cuts in 2009. Romania gains €19.6 billion from EU Structural and Cohesion Funds. Funded by ERDF, the Operational Programme "Increase of economic competitiveness" includes Axis 2: Research, Technological Development and Innovation for competitiveness (€536 m), which is managed through the National Authority for Scientific Research and addresses five of the nine priorities of the national RDI strategy, including (first) 'Health'. The Operational Programme 'Human Resources Development' funded by ESF, has Axis 1 with €797 m for higher education, and includes University education for the knowledge society' and 'Doctoral and postdoctoral programmes in support of research'.

**Slovakia** Strong SF use for research university infrastructures, human resources and programme calls, including clinical research.	Slovakia's proportion of R&D was low at 0.49% by 2007. However, R&D is the main thrust for the 2007-2013 Structural Funds, with €1.2 billion allocated for the Research and Development, €883 m for Convergence, and €326 m for Regional Competitiveness and Employment. Over €500 m was put out to 13 calls in 2009, which included grants for research of around €1 m each (including clinical research studies), for 'centres of excellence' of around €4 m (including environment and health, stroke and perinatology), and grants for SMEs, including several for biomedical technology. The Operational Programme Education, with a total of €617 m, includes Measure 2.1, support for tertiary education (€28 m in 2009 call), as well as Measure 2 2 'Support for life-long learning in the Health sector', with sub-objectives of building human resources for the health system and promoting continuing education (no calls yet under this heading). The Operational Programme, Competitiveness and Economic Growth includes a thematic programme for universities' buildings and infrastructures, with a budget of €1.2 bn

**Slovenia** Moderate SF use for R&D public infrastructures, SMEs and technology. No public health research.	**Slovenia **has a medium level of investment in R&D at 1.6% of GDP in 2008. The total EU Structural Funds are €4.2 billion, divided into five programmes. The first of these, Strengthening Regional Development Potential has €1.7 billion (40%), with five operational programmes, of which the first 'Competitiveness and Research Excellence' receives €402 m (24%). In a competitive call for 2009-2013, eight Centres of Excellence for infrastructures programmes and operation were chosen (out of 60 applications), each receiving €10 m: all were in technology, with one in biochemistry. Seven Competence Centres received €7 m each, with one in biotechnology and one in biomedical engineering.

## Discussion

Understanding of the use of the Structural Funds for health research is limited by the data sources. The European Commission's internet sites provide only high-level information on country research systems [9,10]. The European Commission-funded study of the use of the Structural Funds for health [8] does not cover health research. STEPS Country respondents have described national public health research structures [4], and some more detailed information can be found on country web pages in national languages. This contrasts with information about research available through the European Commission Directorate for Research, which has well-advertised calls and reports at the end of thematic programmes (although the underlying databases remain difficult to interrogate [15]).

The budget available for research through the Structural Funds appears, at least on paper, to be equal in size to the total available for the EU's Research Programme. But the two programmes are managed separately by two European Commission directorates. The Framework Research Programme has a system of National Focal Points, often members of the national research funding agency, but they do not disseminate information about research through the Structural Funds; and ministries of science and education did not show how the Structural Funds contribute to their budgets. This may be because ministries of finance have to report co-funding for the Structural Funds to the European Commission: in general, member states 'badge' the funding as national, so it is impossible to determine how much the Structural Funds themselves have assisted. Our study found that research call programmes were being managed in some countries by the Structural Funds agency of the Ministry of Finance, rather than by the national research agency, creating a 'parallel' system.

While public health research is not prioritised independently within the Structural Funds for research infrastructures, human capacities or research programmes, in Cyprus, Latvia and Lithuania public health appeared to be supported within broader health research. In other countries it is possible that some generic support comes through increased ministry of education funding to universities: but this contrasts with the direct mention in many of the Operation Programmes of funding for engineering and technical sciences, and indeed biotechnology for health sciences. Public health research could compete if calls are developed appropriately, and decision-making boards have the appropriate representation for decision-making. Lithuania appears to have taken this forward with a programme for public health research, although the fields now covered could be widened further.

An underlying problem of priorities is the European Union's priority of support for business [11]. Research and innovation are beneficial for economic 'growth'; yet manufacturing is only a small part of the total of western European economies, and more than 60% is now 'services'. Innovation through human resources and technologies can be social as well as physical, and outside the factory skills, quality and achievements are determined as much by social as by technological factors. This is particularly the case for public health, where national policy, professional practice and scientific knowledge interact, and implementation requires internalisation within cultures beyond commercial markets. There is a need to re-emphasise the importance of not-for-profit research in science [16,17].

Public health research could benefit substantially from the Structural Funds. New institutes, research units and courses could develop within the expanding higher education sector. The research activities do not require expensive laboratories and technical equipment (apart from computing). And public health is a field with a positive gender balance. The challenge is for ministries of health - which would benefit importantly from investment in public health research - to recognise the opportunity. For the Structural Funds in 2007-2013, ministries of health put forward cases for support for both health systems and buildings - and were generally successful, with specific Operational Programmes in most countries [7]. But, despite the high importance of health in research agendas - in European and most national programmes - few ministries of health have internal structures that take a direct interest in research, and have not influenced the priorities of the Structural Funds (through their national ministries of finance). This situation is exacerbated by the historic links of the life sciences with ministries of science rather than ministries of health, and the traditional preference of science academies for basic and 'physical' sciences rather than applied and 'social' sciences.

The content of the EU programmes for 2014-2020 will be decided in 2012 and 2013 through decisions between the Council of Ministers (member states), Parliament (political processes) and European Commission (assessments and implementation). The Structural Funds remain a major part of the EU budget, after the Common Agricultural Policy. In the present funding period, the Structural Funds spend as much in support of research as the EU's official budget for research, yet without any of the controls, data and policies that are needed to improve value. Moreover, the European Commission's Directorate for Regions which allocates and monitors the Structural Funds does not give priority attention to research, and neither the Commission's Directorate for Research nor the Directorate for Health have information on how member states are using Structural Funds for research.

## Competing interests

The authors declare that they have no competing interests.

## References

[B1] Conceição C Leandro A McCarthy M National support to public health research: a survey of European ministries BMC Publ Health 2009 9 203 (25 June) 10.1186/1471-2458-9-203 PMC2713230 19555492

[B2] McCarthy M Clarke A European public health research literatures - measuring progress Eur J Public Health 2007 17 Suppl 1 2 5 17666412 10.1093/eurpub/ckm071

[B3] STEPS country workshops http://www.steps-ph.eu/country-workshop-reports/

[B4] STEPS country profiles http://www.steps-ph.eu/country-research-profiles/

[B5] Conceição C McCarthy M Public health research systems in the European Union Health Res Policy Sys 2011 9 38 10.1186/1478-4505-9-38 PMC3215171 21970897

[B6] Commission E Better health care for Europe and beyond 2010 Brussels: Directorate for Research and Innovation (Public Health Sector)

[B7] McCarthy M Health and the European Structural Funds in the new member states Eur J Public Health 2011 doi 10.1093:eurpub/ckr011 10.1093/eurpub/ckr011 21398381

[B8] Watson J Health and Structural Funds in 2007-2013: Country and regional assessment http://ec.europa.eu/health/health_structural_funds/docs/watson_report.pdf

[B9] Erawatch. Welcome http://erawatch.jrc.ec.europa.eu/

[B10] European Commission Pro-Inno Europe. Inno Policy TrendChart http://proinno.intrasoft.be/index.cfm?fuseaction=page.display&topicID=261&parentID=52

[B11] European Commission Regional Policy Inforegio: Purpose http://ec.europa.eu/regional_policy/what/index_en.cfm

[B12] European Commission: Regional Policy - Inforegio: Three objectives http://ec.europa.eu/regional_policy/how/index_en.cfm

[B13] European Commission: Inforegio: Research and Innovation http://ec.europa.eu/regional_policy/activity/research/index_en.cfm

[B14] European Commission: Regional Policy Inforegio: Project examples http://ec.europa.eu/regional_policy/indexes/project_examples_en.cfm

[B15] Ernst K Irwin R Galsworthy M McKee M Charlesworth K Wismar M Difficulties of tracing health research funded by the European Union J Health Serv Res Policy 2010 15 133 136 10.1258/jhsrp.2010.009115 20466756

[B16] McCarthy M Health research - Europe's future Lancet 2011 377 541 2 10.1016/S0140-6736(11)60184-0 21601702

[B17] Mirowski P Science-Mart: privatising American science 2011 Harvard University Press: London UK

